# Does the Macaque Monkey Provide a Good Model for Studying Human Executive Control? A Comparative Behavioral Study of Task Switching

**DOI:** 10.1371/journal.pone.0021489

**Published:** 2011-06-24

**Authors:** Luana Caselli, Leonardo Chelazzi

**Affiliations:** 1 Department of Neurological, Neuropsychological, Morphological and Motor Sciences, Section of Physiology and Psychology, University of Verona, Verona, Italy; 2 Department of Neurosciences, Section of Physiology, University of Parma, Parma, Italy; 3 Italian Institute of Neuroscience, Verona, Italy; Université Pierre et Marie Curie, France

## Abstract

The ability to swiftly and smoothly switch from one task set to another is central to intelligent behavior, because it allows an organism to flexibly adapt to ever changing environmental conditions and internal needs. For this reason, researchers interested in executive control processes have often relied on task-switching paradigms as powerful tools to uncover the underlying cognitive and brain architecture. In order to gather fundamental information at the single-cell level, it would be greatly helpful to demonstrate that non-human primates, especially the macaque monkey, share with us similar behavioral manifestations of task-switching and therefore, in all likelihood, similar underlying brain mechanisms. Unfortunately, prior attempts have provided negative results (e.g., Stoet & Snyder, 2003b), in that it was reported that macaques do not show the typical signature of task-switching operations at the behavioral level, represented by switch costs. If confirmed, this would indicate that the macaque cannot be used as a model approach to explore human executive control mechanisms by means of task-switching paradigms. We have therefore decided to re-explore this issue, by conducting a comparative experiment on a group of human participants and two macaque monkeys, whereby we measured and compared performance costs linked to task switching and resistance to interference across the two species. Contrary to what previously reported, we found that both species display robust task switching costs, thus supporting the claim that macaque monkeys provide an exquisitely suitable model to study the brain mechanisms responsible for maintaining and switching task sets.

## Introduction

Due to the close proximity in evolutionary terms between the two species, non-human primates are typically taken as almost ideal models for studying cognitive functions of humans. Macaque monkeys, in particular, have been widely used especially in electrophysiological, functional neuroimaging and lesion studies aiming to investigate the neural correlates of cognitive abilities expressed by animals with psychological and behavioral repertoires similar to those of humans. A few fMRI studies have even compared animal and human cognitive mechanisms directly by measuring brain activations in both species while the respective individuals were engaged in performing the same behavioral tasks (e.g., [1]). Therefore, it appears that non-human primates have provided a viable and valuable animal model for exploring several aspects of non-verbal human cognition, including perception, attention, memory, decision making, emotional-motivational processing, and action planning, as well as the underlying brain mechanisms.

Over the past several years, one of the major challenges within human cognitive neuroscience has been to shed light on the functioning and neural substrates of executive control processes. Psychological and neurobiological conceptualizations refer to the executive control system as an overarching system that coordinates cognitive resources for flexibly adapting behavior to immediate environmental demands and moment-to-moment needs of the individual [2]–[8].

Switching at will from one cognitive task to another and the consequent ability to rapidly select the appropriate course of action by favoring task-relevant information in the face of interfering distraction are fundamental components and manifestations of executive control. It is precisely for this reason that task-switching behavioral paradigms have become powerful and widely used experimental tools for obtaining reliable operational measures of such executive functions [9]–[14]. In typical task-switching experiments, subjects perform blocks of mixed-task trials in which they are instructed to switch randomly (or sometimes regularly) between two or more different task rules, represented by competing sets of pre-learned stimulus-response (S-R) associations (task sets). Disengaging from, and preparing for, a particular task set, as well as selecting task-relevant from task-irrelevant information, both represent complex control operations which take time and resources to the system and, consequently, can be detected in the speed and accuracy of behavioral performance, in the form of task-switching and interference costs. These costs are operationally defined as the difference between the levels of performance on two differently demanding behavioral conditions. Three types of task-switching costs have been characterized. *General cost* refers to the increment in reaction times and error rate on mixed-task blocks relative to blocks of trials in which only one task is performed (single-task blocks) [11], [15]–[19]. *Mixing cost* represents a significant fraction of the previously defined general cost and can be measured by specifically contrasting performance on single-task trial blocks against performance on one particular class of mixed-task trials, namely task-repeat trials. By definition, mixed-task blocks include trials in which the task switches relative to the previous trial (task-switch trials) and trials in which the task is repeated (task-repeat trials). Mixing cost quantifies the observation that performance on task-repeat trials, though improving rapidly after a switch, never reaches the level of speed and accuracy of single-task trials [20]. Remarkably, reaction time and error rate decrease substantially on the first task-repeat trial, whereas no additional improvement is detected over subsequent repetitions [11]. *Switch cost* is a trial-specific effect and refers to the worsening in performance associated with changing versus repeating the task executed on the previous trial within mixed-task blocks [11], [12], [17]. It has been sometimes argued that these behavioral effects presumably reflect distinct levels and components of cognitive control involved in task-switching operations [14], [21]. On the one hand, general and mixing costs might index sustained control processes associated with keeping multiple task sets active at the same time, or the division of general cognitive resources between concurrently active task sets. On the other hand, switch costs may be informative about transient executive processes associated with the attentive monitoring of cues signaling task change or maintenance and the rapid updating of current goals.

As a well separate notion, *interference cost* refers to the competition exerted by task-irrelevant information against the selection of task-relevant information. In some task-switching designs, this cost can be operationally measured by comparing performance in relation to congruent versus incongruent bivalent stimuli. Bivalent (or two-dimensional) stimuli are generally assigned with two task set-specific meanings (e.g., colored oriented bars, with color and orientation features prompting two alternative rules), which can be congruent or incongruent in terms of the response required to the subject. Behavioral studies reporting human interference cost have shown that the selection of the currently task-relevant stimulus feature is commonly more effortful (leading to longer reaction times and higher error rates) on incongruent than congruent trials (congruency effect) [10]–[12].

Although the role of prefrontal cortex in central executive functions is of unquestioned importance, the brain circuitry mediating control processes specifically involved in task-switching remains incompletely understood. To date a number of functional neuroimaging studies of executive control have focused on the neural substrates of task-switching and interference behavioral effects in humans [21]–[25]. In contrast, this specific issue has never been addressed in non-human primates, including the macaque monkey, since so far no suitable animal model for studying human task-switching has ever been described in this species. As a consequence, there are no reports in the literature of single-unit recording data in the behaving monkey, which instead would provide fine-scale information regarding the patterns of neural activity associated with the maintenance and switching of task sets.

Indeed, some aspects of executive control closely relevant to task-switching, such as perceptual categorization, associative learning, abstract rule representation and behavioral flexibility, have already been explored in non-human primates using single-unit recording and lesion methods [1], [26]–[33], which indicates that these approaches can be particularly valuable for investigating human high-order cognitive control functions. More importantly, one comparative behavioral study has recently tackled the question whether the macaque monkey can also provide an appropriate model for human task-switching processes [34]. In the reported experiment, both macaques and humans had to randomly switch between two visual discrimination tasks, which were performed on a set of congruent and incongruent bivalent stimuli. The authors reported a modest degree of overlap in behavioral performance costs between the two species. Monkeys showed little or no switch cost and high interference cost, while the opposite pattern was found in humans. This finding would suggest that monkeys radically differ from humans with respect to at least those cognitive processes, and related brain mechanisms, which are responsible for changing task set and maintaining the focus of attention on currently relevant stimulus features. With the present study, we intended to explore further task-switching and interference costs of human and non-human primates so as to reveal inter-species behavioral differences and similarities, which might eventually settle the question whether macaque monkeys are suitable models for studying human executive control and its underlying brain mechanisms. To anticipate, by comparing performance of human and macaque subjects, we obtained evidence that both species show robust task switching costs, therefore supporting the claim that macaque monkeys provide an exquisitely suitable model to study the brain mechanisms responsible for maintaining and switching task sets.

## Methods

### The present study is reported according to the ARRIVE guidelines on animal research

#### Participants

Two adult male Rhesus monkeys (*Macaca Mulatta*, 10 and 8 years of age, weighing about 10 Kg) and eight adult humans (1 male, between 26 and 32 years of age) took part in the study. Use of the macaque monkeys was approved by the University of Verona Committee for Animal Research (CIRSAL) and by the Department for the Veterinary Public Health, Nutrition and Food Security of the Italian Ministry of Health (D.L. n. 116/1992, art. 8/9 and D.M. n. 53/2003-c, 04/04/2003). The monkeys were housed and handled in strict accordance with the Weatherall Report's recommendations about good animal practice and their wellbeing and health conditions were constantly monitored by the institutional veterinary doctor.

A scleral search coil for eye position recording and a head-restraint device for painless immobilization of the head were implanted under aseptic conditions while the monkeys were anesthetized (Domitor medetomidina 1 mg/ml, Orion Pharma, Espoo, Finland). The veterinary doctor assisted the surgical operation and closely monitored the animals both during surgery and in the following days. Experiments resumed two weeks or the time necessary after postoperative analgesics and antibiotics administration.

The monkeys were habituated to the experimental routine and the experimenter. While participating in this study, monkeys were on water restriction and performed the experiment in exchange for liquid reward, along the lines of operant conditioning. A minimum daily level of fluid intake for adequate hydration was determined separately for each animal and, if necessary, the amount of water or juice acquired during the experimental session was supplemented by additional fluid upon returning the animal to the home cage. Moreover, the body weight of the monkeys was monitored daily to exclude excessive weight loss. These procedures were closely supervised by our veterinary doctor.

#### Apparatus and stimuli

**Figure 1:**
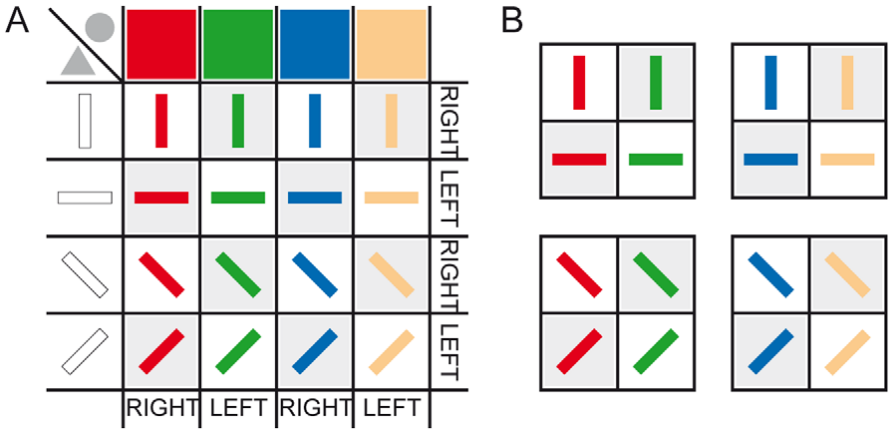
Tables of S-R contingencies. A. Stimuli on gray background are incongruent; stimuli on white background are congruent; gray circle and triangle are the symbolic cues instructing the color and orientation task, respectively. B. Subsets of stimuli used in monkey sessions.

Behavioral paradigm administration and data acquisition were monitored by a computer running the “CORTEX" real-time control system (http://www.cortex.salk.edu/). Experimental sessions took place in a sound-attenuated, dark room. Stimuli were presented onto a computer screen positioned 57 cm in front of the participants. Two different feature-discrimination tasks were performed on a set of target stimuli consisting of sixteen colored oriented-bars (2.9°×0.5°), resulting from the combination of four colors (red, green, blue and yellow) and four orientations (vertical, horizontal and the two orthogonal oblique orientations). Each feature value was arbitrarily associated to either of two alternative motor responses (the turning of a response lever in the clockwise or counter-clockwise direction), according to the stimulus-response (S-R) contingencies shown in Figure 1a. In the case of congruent stimuli, both the color and the orientation of the stimulus were associated to the same motor response, whereas in the case of incongruent stimuli, color and orientation features of the given stimulus were associated to incompatible responses. Either of two different symbolic cues informed the subject about which task was to be performed on the upcoming target stimulus.

Animals were seated in primate chairs and a juice spout was placed next to their mouth for automated reward delivery in return of correct behavioral performance. Both monkeys and humans received acoustic feedback after correctly performed trials.

#### Procedure

All subjects performed a cued task-switching paradigm. At the beginning of each trial, either a color-task cue (a circle) or an orientation-task cue (a triangle) instructed the subject as to which component feature of the upcoming target stimulus had to be discriminated while disregarding the other feature of the same stimulus. Consequently, the task cue indicated which of the two pre-learned sets of S-R associations had to be implemented on the basis of the specified feature. After stimulus onset, subjects had to turn the lever according to the response required by the currently attended feature value. Obviously, before running the experimental sessions, monkeys and humans had experienced different amounts of practice on the relevant S-R mappings. Monkeys had received several training sessions on each of four different subsets of the sixteen target stimuli (Figure 1b), while humans, after being verbally instructed, experienced only one short block of practice trials. Also, unlike what we did for the monkeys, we presented humans with the entire set of target stimuli in order to increase the number of combinations and thus encourage them to apply general feature-based strategies rather than specific S-R associations. Also during each experimental session with monkeys, behavioral data were collected by using one randomly selected subset of stimuli, while human subjects always faced all the 16 stimuli.

Color- and orientation-discrimination trials were presented in both single- and mixed-task sequences. In single-task sequences, the participants were required to perform the same task for twenty-four consecutive trials before switching to the other, competing task. On the contrary, in mixed-task sequences, the feature guiding the selection of the motor response might change on a trial-by-trial basis, thus including both trials in which subjects had to switch from one task to the other (task-switch trials) and trials in which they continued performing the same task (task-repeat trials). Although not considered for statistical analyses, trials in which monkeys broke eye fixation at any time after cue offset (i.e., when the animal had already received the task instruction) were discarded only after having sorted mixed-task trials into “task-switch" or “task-repeat" classes.

**Figure 2:**
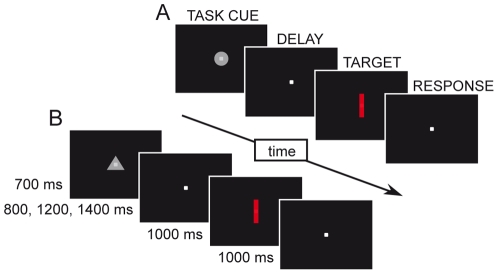
Task design. A. Example of color-discrimination trial. B. Example of orientation-discrimination trial.

After an initial 700 ms interval during which central fixation was required, the task cue appeared and remained visible for 700 ms. Then, subsequent to a randomly variable cue-stimulus period (800, 1200 or 1600 ms), the target stimulus was presented for 1000 ms or until the behavioral response. The task cue and target stimuli were always presented at the fixation point location (Figure 2). The inter-trial interval lasted 3 seconds. To note that while in the monkeys eye fixation was monitored by means of the scleral search coil method, no direct measure of eye fixation was taken from the human participants, and they were simply asked to maintain their gaze on the central fixation point for the entire duration of each trial.

#### Statistical analyses

Task-switching costs and interference costs were analyzed separately for humans, M1 and M2 by performing 3-way repeated measures ANalyses Of VAriance (ANOVA) on both error rate (ER) and reaction time (RT) data. A 2×2×2 ANOVA with the factors *sequence* (single-task, mixed-task), *task* (color, orientation) and *stimulus congruency* (congruent, incongruent) was used to examine general costs. A 3×2×2 ANOVA with the factors *trial type* (single-task, task-repeat, task-switch), *task* and *stimulus congruency* was applied to analyze mixing and switch costs. Interference costs were evaluated by examining the main effect of the factor *stimulus congruency*. We used paired samples T tests for post-hoc statistical evaluation.

## Results

Results obtained from Monkey #1 (M1), Monkey #2 (M2) and the human participants will be described separately, and they refer to behavioral data collected during 82, 40 and 8 experimental sessions, respectively. Data acquired from each human participant were collected during a single experimental session.

**Figure 3:**
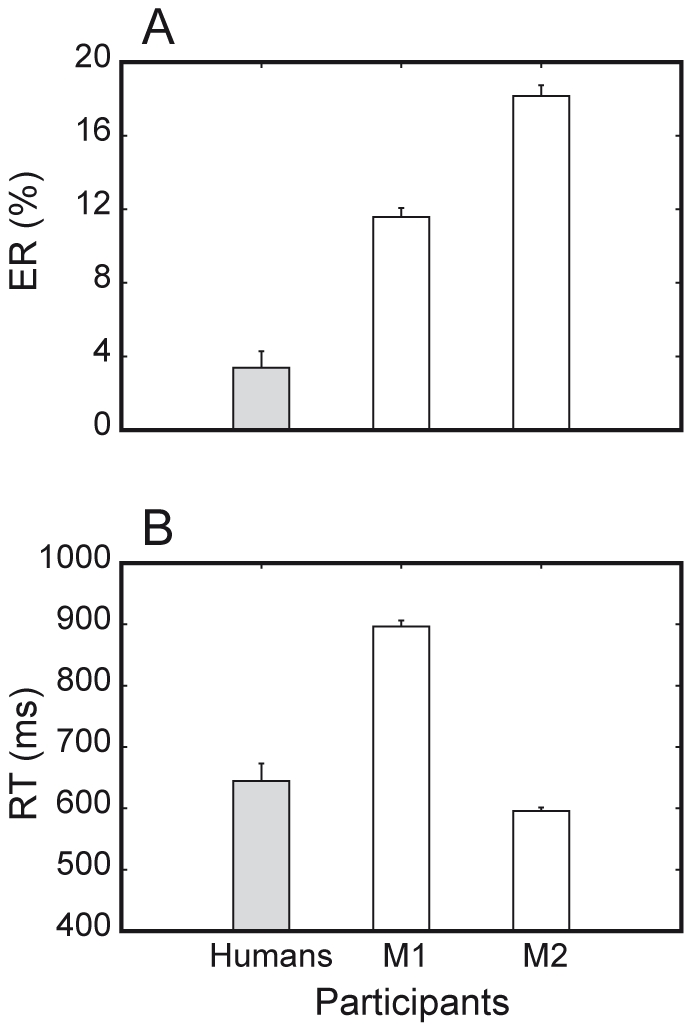
Overall behavioral performance of humans and monkeys. A. Overall error rate. Humans (gray) are significantly more accurate than monkeys (white). M1 is more accurate than M2 (differences significant at P<0.0001). B. Overall RTs. Humans and M2 are faster than M1 (P<0.0001).

As a first step, we looked for any inter-species difference in overall behavioral performance by comparing error rate (ER) and reaction times (RTs) of humans, M1 and M2 across all trials through a one-way ANOVA (Figure 3). Humans were generally more accurate than both M1 and M2 (mean ER = 3.4% versus 11.6% and 18.2%, respectively; differences significant at P<0.0001); humans were also significantly faster than M1 (mean RT = 644 ms versus 896 ms; P<0.0001) but slower than M2 (mean RT = 644 ms versus 596 ms; P<0.01). In addition, M1 was significantly more accurate but slower than M2 (mean ER = 11.6% versus 18.2%, mean RT = 896 ms versus 596 ms; differences significant at P<0.0001), perhaps reflecting a form of speed-accuracy tradeoff in performance between the two animals.

**Figure 4:**
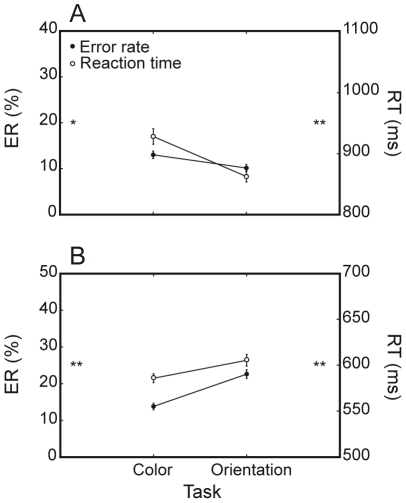
Error rate and RT of monkeys on color- and orientation-task trials. Monkeys perform the two tasks at a different level of accuracy and speed. A. M1 is less accurate and fast on color-task trials. B. M2 is less accurate and fast on orientation-task trials. [* = P<0.05; ** = P<0.0001].

Given this general behavioral pattern, we then examined species-specific task-switching and interference costs, both in terms of ER and RTs by performing the statistical analyses described in the Methods section (table 1 summarizes the main behavioral effects). Both monkeys performed the two tasks at different levels of accuracy and speed (Figure 4). M1 was more accurate and faster on orientation- than on color-discrimination trials (ER: 10.1% versus 13%, F(1, 81) = 5.1, P<0.05; RT: 864 ms versus 929 ms, F(1, 81) = 48.8, P<0.0001; Figure 4A), while M2 performed better on color- than on orientation-discrimination trials (ER: 13.8% versus 22.6%, F(1, 39) = 25.1, P<0.0001; RT: 586 ms versus 606 ms, F(1, 39) = 27.2, P<0.0001; Figure 4B). Since these differences did not interact with any other factor in a substantial and reliable fashion, behavioral costs related to congruency and task switching effects for both monkeys are reported after collapsing data from the two tasks.

**Table 1:** Task switching costs of humans, M1 and M2.

	Interference costs		General costs		Mixing costs		Switch costs		1^st^ rep–2^nd^ rep	
	Errors (%)	RTs (ms)	Errors (%)	RTs (ms)	Errors (%)	RTs (ms)	Errors (%)	RTs (ms)	Errors (%)	RTs (ms)
**Humans**	2.6	14*	2.7	67	1*	51	3.2	29	2.5	27*
**M1**	18.5	109	4.7	17	3.9	16	4.3	16	3.1	6*
**M2**	27.3	57	2.9	2*	0.6*	1.5*	5.6	−0.2*	4.1	−2*

*Statistically not significant.

### 

#### Interference costs

**Figure 5:**
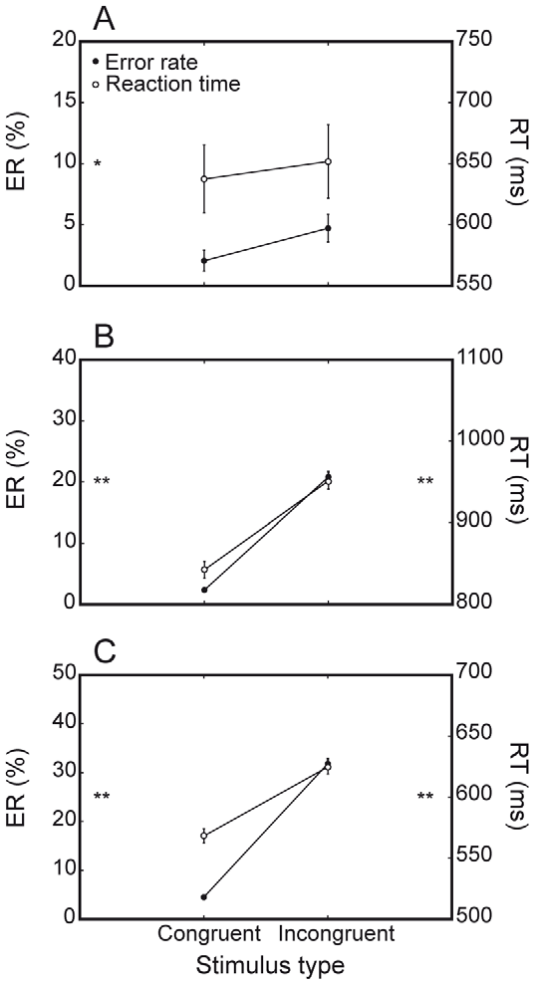
Interference costs. Both species display consistent interference costs. A. Humans are significantly less accurate on incongruent than congruent trials. B. M1 is significantly less accurate and fast on incongruent than congruent trials. (c) M2 is significantly less accurate and fast on incongruent than congruent trials. [* = P<0.05; ** = P<0.0001].

Both humans and monkeys showed significant interference costs (Figure 5). The congruency effect was striking for M1 and M2, both in terms of ER difference between congruent and incongruent trials (18.5%, F(1, 81) = 399.7, P<0.0001, and 27.3%, F(1, 39) = 487.9, P<0.0001, respectively, for the two animals) and RT difference between the same two conditions (109 ms, F(1, 81) = 489.9, P<0.0001, and 57 ms, F(1, 39) = 176.7, P<0.0001, respectively, for the two animals). Human participants displayed a less dramatic but nonetheless consistent interference cost in terms of ER (2.6%, F(1, 7) = 8.7, P<0.05), but were not significantly faster on congruent than incongruent trials (637 ms versus 651 ms, P = 0.2). Interestingly, the magnitude of interference costs in the form of ER differences appeared to be proportional to the species-specific overall accuracy levels reported above: human participants, who were overall more accurate than monkeys, displayed lower congruency effects with respect to both M1 and M2. Similarly, M2, whose general accuracy level was comparatively poorer, paid the highest error interference cost (differences significant at P<0.0001).

#### General costs

**Figure 6:**
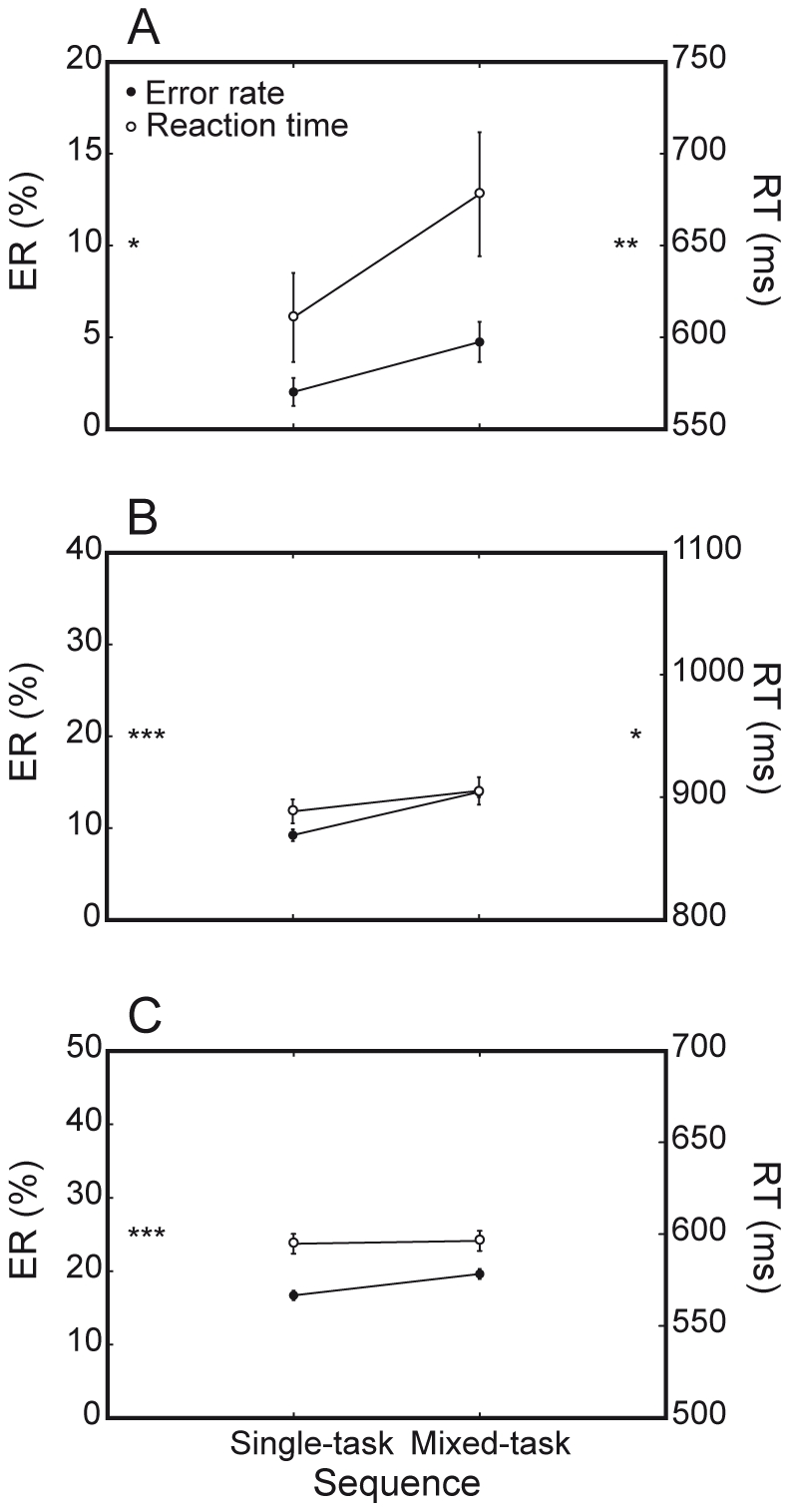
General costs. Both species show significant general costs. A. Humans are significantly less accurate and fast on mixed-task than single-task trials. B. M1 is significantly less accurate and fast on mixed-task than single-task trials. C. M2 is significantly less accurate on mixed-task than single-task trials. [* = P<0.05; ** = P<0.005; *** = P<0.0001].

Both humans and monkeys showed highly significant general costs in terms of ER, as previously defined (humans: 2.7%, F(1, 7) = 18.7, P<0.01; M1: 4.7%, F(1, 81) = 46,7, P<0.0001; M2: 2.9%, F(1, 39) = 46.7, P<0.0001; Figure 6). Note that, unlike interference costs, and despite dissimilarity between species in overall accuracy, these ER costs were comparable (F(2, 127) = 1.7, P = 0.2). General cost in terms of RT differences was significantly displayed by humans (67 ms, F(1, 7) = 19.9, P<0.005) and M1 (17 ms, F(1, 81) = 6,9, P<0.01). M1 general cost on RTs mainly concerned orientation-discrimination trials, as expressed by the significant *sequence* by *task* interaction (F(1, 81) = 11.9, P = 0.001) obtained for this animal. On the contrary, M2 was not consistently faster on single-task compared to mixed-task trial sequences (mean RT general cost = 2 ms, F(1, 39) = 0.7, P = 0.4).

**Figure 7:**
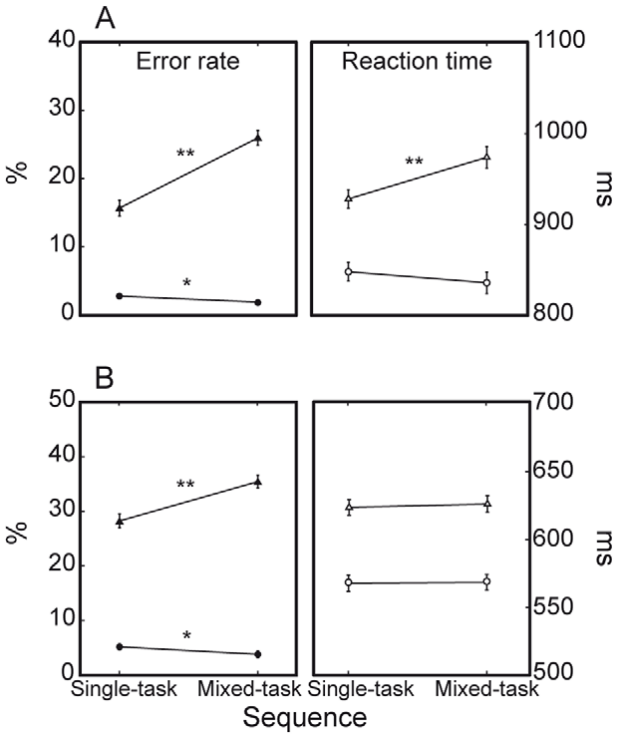
Interaction between interference costs and general costs. Monkeys show significant sequence by stimulus congruency interactions. A. ER and RT general costs of M1 are higher on incongruent (triangles) than congruent (circles) trials. B. ER general cost of M2 is higher on incongruent trials. [* = P<0.05; ** = P<0.0001].

Remarkably, general and interference costs shown by monkeys significantly interacted, especially when looking at accuracy (M1: F(1, 81) = 58.3, P<0.0001; M2: F(1, 39) = 48.0, P<0.0001). Specifically, ER general costs for both M1 and M2 emerged robustly on incongruent stimuli while ER on congruent stimuli was even slightly higher on single-task blocks compared to mixed-task blocks (Figure 7). M1 also showed a significant interaction between general and interference costs in RTs, revealing a pattern of results consistent with that just described for ER (F(1, 81) = 52.6, P<0.0001).

#### Mixing and switch costs

**Figure 8:**
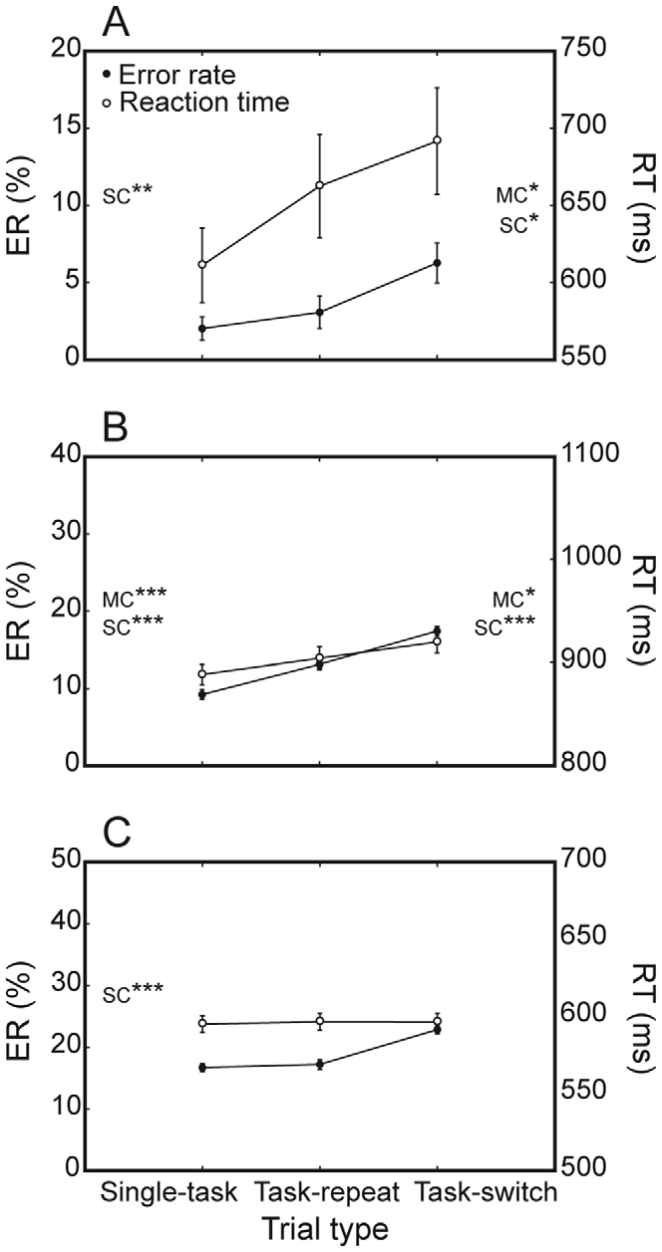
Switch and mixing costs. Both species show significant switch costs (SC) and mixing costs (MC). A. Humans are significantly less accurate and fast on task-switch than task-repeat trials (switch cost). They are also less fast on task-repeat than on single-task trials (RT mixing cost). B. M1 displays switch and mixing costs, in terms of both accuracy and speed C. M2 shows significant ER switch cost. [* = P<0.05; ** = P<0.001; *** = P<0.0001].

Mixing and switch costs, which are specifically related to the difference in performance across different trial types (single-task, task-repeat and task-switch trials) were statistically evident as significant *trial type* main effects in both humans (ER: F(2, 7) = 17.6, P<0.001; RTs: F(2, 7) = 16.8, P<0.001) and monkeys (M1: ER, F(2, 81) = 88.0, P<0.0001; RTs, F(2, 81) = 15.9, P<0.0001; M2: ER, F(2, 39) = 52.9, P<0.0001; RTs, F(2, 39) = 189, P = 0.8, n.s.). These task switching costs are shown in Figure 8.

Paired T-test comparisons revealed that mixing cost was expressed by humans as a significant RT difference between task-repeat and single-task trials (51 ms, P<0.05). M1 showed a reliable mixing cost both in terms of ER (3.9%, P<0.0001) and RTs (16 ms, P<0.05). RT mixing cost for this monkey was particularly robust on orientation discrimination trials (34 ms; significant *trial type* by *task* interaction, F(2, 81) = 7.5, P<0.01). Moreover, as already reported for other results, responses of M1 to congruent stimuli were curiously faster on task-repeat than on single-task trials (834 ms versus 848 ms, P<0.05). M2 had no mixing cost at all.

Switch cost in ER was displayed by humans (3.2%, P<0.01), M1 (4.3%, P<0.0001) and M2 (5.6%, P<0.0001). As in the case of general costs, ER switch costs of monkeys were comparable to those of humans (F(2, 127) = 1.9, P = 0.1). Switch cost in RTs was observed only in humans (29 ms, P<0.05) and M1 (16 ms, P<0.0001).

**Figure 9:**
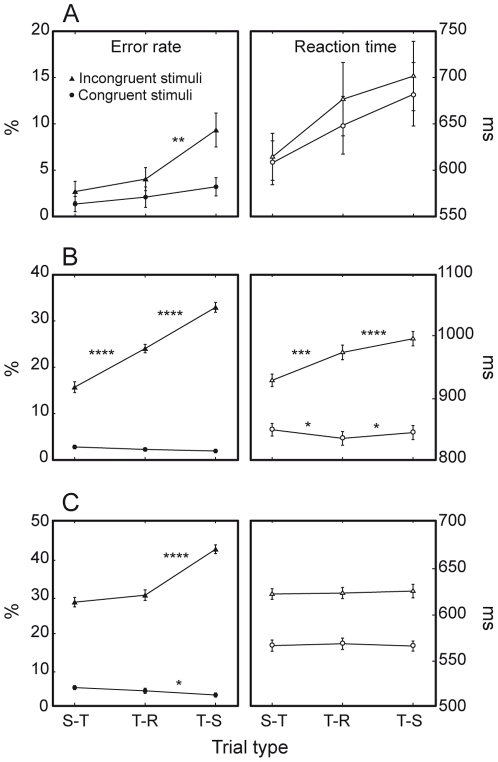
Effects of interference costs on mixing and switch costs. Monkeys show significant trial type by stimulus congruency interactions. A. ER switch cost of humans is higher on incongruent (triangles) than congruent (circles) trials. B. ER and RT mixing and switch costs of M1 are higher on incongruent trials. C. ER switch cost of M2 is higher on incongruent trials. [* = P<0.05; ** = P<0.01; *** = P<0.001; **** = P<0.0001].

Similarly to general costs, switch costs depended on the response conflict posed by the given stimuli (Figure 9). All subjects were generally less accurate when required to change task set on incongruent trials, as expressed by the significant *trial type* by *stimulus congruency* interaction (humans: F(2, 7) = 6.6, P<0.01); M1: (F(2, 81) = 96.2, P<0.0001); M2: (F(2, 39) = 86.5, P<0.0001). Furthermore, only M1 showed an increased switch cost for incongruent trials in the form of slowed RTs (F(2, 81) = 51.1, P<0.0001).

#### Task repetition effect

**Figure 10:**
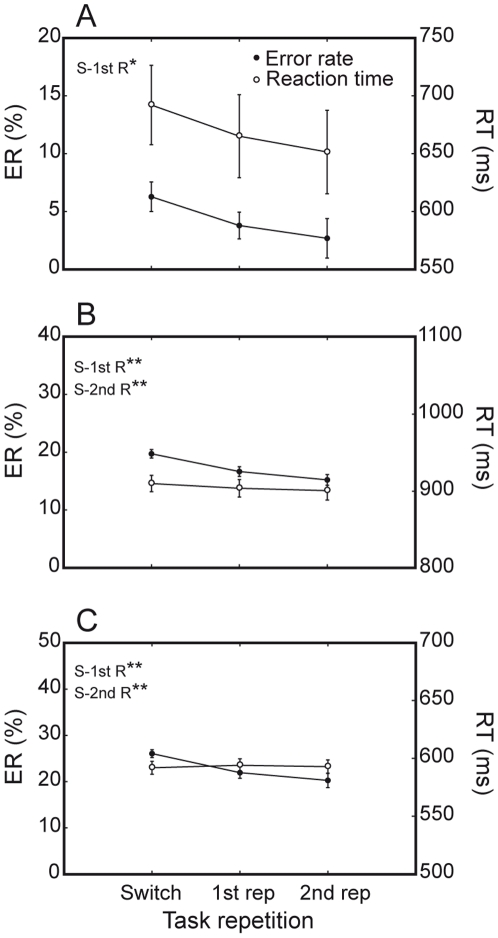
Task repetition effect. Error rate and RTs of humans and monkeys on switch (S), first task-repeat (1^st^ R) and second task-repeat (2^nd^ R) trials. Both species substantially improve their performance from the switch trial to the first of a series of task-repeat trials. (A) Humans; (B) M1; (C) M2. [* = P<0.05; ** = P<0.0001].

A repeated measures ANOVA with the factors *task* and *trial repetition* (1, 2 and 3, with 1 designating a task switching trial) run on data collected on mixed-task sequences showed a highly consistent effect of task repetition in ER (humans: F(2, 7) = 4.3, P<0.05; M1: F(2, 81) = 13.6, P<0.0001; M2: F(2, 39) = 9.2, P<0.0001) (Figure 10). In particular, the accuracy of both humans and monkeys improved substantially from the switch trial to the first of a sequence of task-repeat trials but did not significantly improve thereafter (differences significant at P<0.05 for humans and at P<0.0001 for M1 and M2). Although not significantly so, also reaction times, at least those of humans and M1, became generally shorter on the first post-switch trial, while they tended to remain stable thereafter.

## Discussion

The ability to swiftly and smoothly switch from one task set to another requires high-level cognitive functions that are at the core of executive control, and is a hallmark of intelligent behavior. An animal model expressing such functions would be extremely valuable for studying the neural basis of human complex behavior, including at the cell-physiological level. In order to find out whether non-human primates can approach this model, we compared human and macaque monkey behavioral performance on a task-switching paradigm.

A first remarkable result that we obtained pertains to overall performance. We found that humans are generally more accurate than macaques, probably suggesting that the paradigm we used requires different levels of cognitive effort by the two species or, in different words, that it challenges their cognitive capabilities to a different degree. Moreover, although overall higher error rates in M2 may perhaps be explained by a speed-accuracy tradeoff in this monkey (M2 was less accurate and faster than both M1 and humans), the fact that M1 was both slower and less accurate than humans supports the conclusion that the task was more difficult for the monkeys than for the human participants, presumably reflecting a difference in the amount of species-specific cognitive resources. Task-switching and interference costs were thus computed, allowing us to more directly reveal any interspecies differences in critical components of central executive functions, which in turn might be related to the above basic behavioral dissimilarity.

Both species showed consistent interference costs in terms of reduced accuracy for incongruent versus congruent conditions, indicating that an inability to completely disable information that is irrelevant to current goals might represent a critical limitation of primate executive control. However, monkeys manifested these costs more markedly than humans, and also their response times increased to a greater extent when processing stimuli associated with two incompatible S-R mappings. This second-order behavioral result, in conjunction with the observation that macaque monkeys are generally more inaccurate than humans, suggests that non-human primates have less control on selecting and pursuing a specific course of action which is appropriate to the task at hand.

Performance effects typical of task-switching were detected in both species; M1 and to some extent also M2 expressed behavioral costs comparable to those observed in humans. Humans and macaques paid similar costs if asked to perform two alternative tasks along the same trial sequence rather than one and the same task for many consecutive trials (general cost). Except for reaction times of M2, which were not particularly affected by the type of sequence, general costs shown by both species are indicative of reduced cognitive efficiency resulting from the need to prepare for shifting on request between two different task sets. In particular, worsening of M1 and human performance on task-repeat trials relative to single-task trials (mixing costs) strengthens the notion that the executive system of primates has finite resources: the cognitive load imposed by the mental operation of keeping more than one task set simultaneously active in memory is sufficient to make performance on task-repeat trials decline relative to single-task trials. Indeed, although both monkeys and humans quickly and substantially enhanced their performance immediately after a switch trial, that is on the first task-repeat trial, they were still not so proficient as on single-task blocks and did not further improve as a function of task repetitions.

In addition to the latter costs due to sustained demands on attentional control, producing global performance deficits, we found that the dynamic process of rapid task set re-configuration within a mixed-task block adds transient trial-specific costs in both human and monkey task-switching behavior. We underscore that macaques displayed switch costs that are comparable in size to those paid by humans, suggesting that they share the fundamental limitations in executive control which typically make us humans less accurate and prompt to respond on trials requiring a task switch compared to task-repeat trials. Therefore it appears that switch costs of monkeys, when conveniently characterized, provide a valuable window onto human (and, by extension, primate) transient control components, which are responsible for short-term operations such as preparing the cognitive set specified at each trial start by the instructional cue (endogenous control component) and completing task set implementation after target stimulus presentation, when all pertinent information has to be extracted in order to finally release the appropriate motor response (exogenous control component) [11].

Within the cognitive psychology literature, it has been demonstrated that task set re-configuration cannot be entirely achieved until exogenously triggered by stimulus attributes that are associated with the current task [11], [12]. In particular, even when advance knowledge is provided and long cue-stimulus intervals are allowed to adequately prepare for the upcoming task prior to stimulus onset, switch RT costs are reduced but not completely abolished. It has been proposed that this *residual cost* likely reflects the time needed for instantiating exogenous control, and in particular for selecting task-relevant stimulus features and retrieving the appropriate S-R associations [11], [35]. The observation that switch costs of both humans and macaques are significantly affected by stimulus congruency appears to support this general hypothesis, suggesting that exogenously triggered executive functions are crucially involved in primate task-switching operations. According to our results, switch costs paid by the two species, especially in terms of increased error rates, are particularly large when target stimuli are associated with two S-R mappings prompting incongruent motor responses. In contrast, switch costs are reduced or non-existent on trials entailing the retrieval of the currently task-relevant association for congruent stimuli (Note 1). This performance difference clearly denotes the incompleteness of reconfiguration at the time of stimulus onset: conflicting S-R mappings should interfere with residual switch costs as much as response-compatible associations, if task set implementation had already been accomplished endogenously. This result thus confirms that stimulus congruity effects not only reveal the persistence of the supposedly disengaged mapping, but may also be informative about the primate exogenous control components which allow selection of task-relevant stimulus features in task-switching.

However, macaques seem to differ from humans at least in the way interference effects interact with task switching-specific behavioral costs. Contrary to humans, M1 and M2 also displayed general costs interacting with stimulus congruency. Moreover, the direction of costs was often peculiarly reversed on congruent trials, i.e. performance was better on the condition which is supposed to be the most difficult. For instance, macaques were paradoxically more accurate or faster on congruent trials of a mixed-task block than on the same congruent trials presented within a single-task condition (see Figure 7). Likewise, the trial type-related pattern of behavior they expressed on congruent trials is quite opposite to expectations. Although differences are small or non-significant, both M1 and M2 tended to perform worse on single-task trials than on task-repeat trials and showed reversed switch costs (see Figure 9). These peculiar results depend in all probability on the basic inter-species difference in overall performance. Since the task-switching paradigm we used exposes macaques to a higher level of difficulty relative to humans, it is likely that especially the mixed-task context elicits in monkeys a state of increased arousal, such that their cognitive efficiency is enhanced compared to the single-task condition. Within this context, it may happen that trials involving congruent S-R mappings, which a priori should be equally demanding within either trial sequence and should not impose any additional load to the monkey executive system during task-switching control operations, are curiously performed better under conditions of elevated cognitive engagement.

In conclusion, macaque monkeys appear to match many aspects of human task-switching, at least at the behavioral level. They pay similar performance costs, revealing common psychological limitations which in both species lead to interference effects on information-selecting operations and lack of cognitive flexibility under contexts requiring dynamic goal-updating processes. However, monkeys appear to be overall less competent than humans in managing task-switching-like situations, suggesting that their executive system is fundamentally more resource-limited. Actually, even if one were to suppose that this represents an important inter-species difference, the magnitude of task-switching-specific behavioral effects is comparable in macaques and humans, indicating that they might depend on analogous cognitive control components, but acting on different efficiency scales.

In contrast to our results, Stoet and Snyder [34], who focused their study on trial-specific task-switching effects, did not report any consistent switch cost in monkeys, suggesting that in these animals, unlike humans, task sets can be completely reconfigured prior to stimulus presentation. The authors of the study reported evidence to suggest that switch costs could be obtained with particularly short inter-trial intervals (170 ms). However, this is an unlikely explanation for the robust switch costs we observed in monkeys, since in the present experiment we used inter-trial intervals of 3 seconds. Moreover, as they pointed out, the increased flexibility they observed in monkeys cannot be a function of training, since it has been demonstrated that humans still pay switch costs after extensive practice sessions [36], [37] and our study shows that switch costs persist even in long-trained monkeys.

We have no simple explanation for this apparent discrepancy in results. Perhaps one possibility relates to a different training strategy between our study and theirs, but at present this is only speculative. It remains that in our hands the macaque monkey provides an excellent model to study human task switching performance, both at the behavioral and neurobiological level. We can actually conceive that, although not necessarily sharing the complex behavioral experiences of human individuals, non-human primates exert cognitive control processes that may well approach the human executive system.

### Notes

#### Note 1

An alternative possibility is simply that congruent stimuli do not provide an adequate test of task switching performance. When a congruent stimulus is presented on the occasion of a task-switch trial, the animal may even fail entirely to switch to the newly relevant task and accordingly select the relevant feature of the stimulus, and yet produce a correct motor response for the simple reason that both features (and therefore both tasks) require the same motor response. In other words, in the case of congruent stimuli it is conceivable that a considerable number of task-switch trials appear to be correct but should in fact be classified as incorrect, had we a way to know that the animal has performed the now irrelevant task.
